# Parental Support during the COVID-19 Pandemic: Friend or Foe? A Moderation Analysis of the Association between Maternal Anxiety and Children’s Stress in Italian Dyads

**DOI:** 10.3390/ijerph20010268

**Published:** 2022-12-24

**Authors:** Alessia Cadamuro, Elisa Bisagno, Elena Trifiletti, Gian Antonio Di Bernardo, Emilio Paolo Visintin

**Affiliations:** 1 Department of Biomedical, Metabolic and Neural Sciences, University of Modena and Reggio Emilia, 41125 Modena, Italy; 2 Surgical, Medical and Dental Department of Morphological Sciences Related to Transplant, Oncology and Regenerative Medicine, University of Modena and Reggio Emilia, 41124 Modena, Italy; 3 Department of Human Sciences, University of Verona, 37129 Verona, Italy; 4 Department of Education and Human Sciences, University of Modena and Reggio Emilia, 42121 Reggio Emilia, Italy; 5 Department of Humanities, University of Ferrara, 44121 Ferrara, Italy

**Keywords:** COVID-19, children’s stress, maternal anxiety, helicopter parenting, parental support

## Abstract

There is evidence that parental psychological disorders in stressful situations increase the risk of disturbance in child development. This has been investigated in disasters but not in pandemics, which are sensibly different from other types of traumatic events. We investigated the relationship between mothers’ anxiety and their children’s (self-reported) stress and the boundary conditions of this association during the first full COVID-19 lockdown in Italy. During the COVID-19 pandemic, mothers might have increased their protective attitudes to secure and support their children; we tested whether the relationship between mothers’ anxiety and children’s stress was weaker (buffer effect) or stronger (over-protection effect) when perceived parental support was high. We measured mothers’ anxiety, children’s perceived parental support, and children’s stress in a sample of 414 8- to 11-year-old primary school children (229 females, Mage = 9.44) and 395 mothers (Mage = 42.84). Results supported the over-protection scenario and provided the first evidence for the “helicopter-parent effect” during the COVID-19 pandemic: mothers’ anxiety was positively associated with children’s stress only when perceived support was high. Our finding highlights the importance of educating parents (for example, via emotional training) to prevent the worst consequences of adverse events in children and promote their mental health.

## 1. Introduction

There is evidence that parental psychological disorders increase the risk of disturbance in child development [1,2] and that parental stress predicts children’s mental and behavioral problems [3]. In particular, maternal stress can be an important factor associated with lower psychological adjustment in children [4,5], while maternal anxiety was identified as the most frequent and important predictor of children’s mental health status [6].

Although the relationship between parents and children’s psychological well-being has been extensively investigated in stressful situations, such as wars and disasters, the same does not hold for pandemics, which are sensibly different from other types of traumatic events [7,8]. The COVID-19 pandemic has determined unprecedentedly strict quarantine measures. During the outbreak, children and their parents were isolated and confined together at home, with substantial changes in their daily routine and social infrastructure. An increasing body of research suggests that being in lockdown is associated with poor social and emotional well-being in both adults [9] and children [10].

The main aim of this study was to investigate whether there is an association between mothers’ anxiety and children’s (self-reported) stress with respect to the COVID-19 pandemic. A related aim was to investigate the boundary conditions of this association by examining the moderating role of parental support. Historically, social support has been considered a protective factor. Specifically, the buffering hypothesis suggests that social support protects (‘‘buffers’’) individuals from the damage caused by stressful events [11]. Based on this hypothesis, we could expect that high perceived maternal support during the pandemic weakened the association between maternal anxiety and children’s stress. However, given the unprecedented characteristics of the pandemic and the subsequent lockdown, we could also formulate the reverse hypothesis, which is that mothers might have developed over-protective attitudes to support their children, thus leading to the counter-intuitive result that perceived maternal support during the pandemic boosted the association between maternal anxiety and children’s stress instead of buffering it. We explored these hypotheses during the first lockdown in a sample of mothers and children aged 8 to 10 years living in Italian regions highly affected by COVID-19.

### 1.1. The Relation between Parents’ Psychological Response to the Pandemic and Children’s Stress

Due to the pandemic, children were reported to suffer psychological problems, such as anxiety and depression [12], resulting in poor cognitive performances [9,10,12,13], which are typical symptoms of post-traumatic stress disorder. A role may have been played by social distancing and lockdown, which removed many of the social, educational, and community social supports (e.g., physical activity) [12] that protect children in times of crisis. In such a situation, parents remain the only source of social support for children. However, the pandemic has had severe effects on adults’ mental health and well-being [14], which might negatively reflect on children.

Research has provided evidence for an association between parental exposure to traumatic stress and children’s mental health issues due to traumatic events. For instance, the relationship between parents’ stressful experiences and psychological symptoms and children’s psychological dysfunctioning has been demonstrated in the case of exposure to war [15,16] or disasters [17,18], revealing that the parental response is a powerful predictor of children’s emergency adjustment [19,20].

The same association may hold as a consequence of a unique event such as a pandemic. A pandemic represents an atypical traumatic event, characterized by different features compared with other types of collective traumatic events, such as disasters. A pandemic disrupts patterns of socialization that significantly contribute to our sense of identity, and it demands social distancing, which can induce distress in the form of a sense of loneliness and feelings of grief and anxiety [14]. Furthermore, a pandemic is not confined to time and space, and the feeling of being “out of control” of what is happening triggers negative emotions, such as fear and anger [21]. Not knowing when it will end and being provided with contradictory indications on which safety measures to follow can be a source of stress [9], while being isolated increases the risk of “transmitting” stress within families [22].

As stated above, parents’ stress may be a relevant antecedent of children’s stress. However, although the relationship between parents and children’s stress is well-established in the literature, only a few studies have investigated it in response to a pandemic and, specifically, to the COVID-19 pandemic. Initial evidence was provided by Spinelli and coworkers [23], who assessed parents’ COVID-19 contact risk (based on the contact with Covid-diagnosed individuals), parents’ stress (regarding both individual stress and stress attributed to the child-parent relation), and 2 to 14 years-old children’s psychological problems. Results revealed that parents’ COVID-19 contact risk was indirectly associated with children’s greater psychological problems via both forms of parents’ stress. Similar results were reported by Lee and coworkers [24], who considered the relationship between parents’ and 0 to 12 years-old children’s stress during the pandemic: parental anxiety and parenting stress were associated with higher levels of child anxiety. Davico and coworkers [25] reported that the levels of Covid-related stress perceived by parents correlated with stress perceived by children aged 8 to 18 years. These studies, however, suffer from some important limitations. Children’s stress was reported by parents [23,24] rather than self-reported; or the instrument was administered to children by parents, with consequential problems of reliability [25]. Therefore, results from these studies suffer from reliability issues and qualify as preliminary evidence regarding the association between parents and children’s stress.

We decided to test the existence of a relationship between the psychological response of parents to the COVID-19 pandemic and the impact of the stressful event on children, using a reliable assessment where children themselves report their stress to a trained researcher. To the extent that maternal anxiety was identified as the most frequent and important predictor of children’s mental health status [6], we studied the relationship between mothers and children, focusing for the first time on the construct of maternal anxiety as a predictor of children’s stress within the context of the COVID-19 pandemic. Based on previous literature on the relationship between parents’ psychological response to stressful events and children’s well-being (see [19], for a review), we also decided to consider another relevant variable that could moderate this association, namely social support. 

### 1.2. Parental Support during the Pandemic

Since 1970, the beneficial role of interpersonal relationships in safeguarding people from the detrimental effects of stressful events has been studied [26]. The term “social support” has been used to describe the mechanisms by which interpersonal relationships reduce the harmful impact of distress on one’s mental health [27]. Specifically, the buffering hypothesis suggests that social support counteracts the effects of stress by either shifting one’s perception of the threat or adjusting one’s stress response via the provision of physical comfort, emotional support, or advice [27]. The buffer effect has been considered a key determinant in stress regulation throughout the lifespan, with a critical role played by parents for their children [28]. Moreover, this effect has been found in a variety of stressful situations, from expected life transitions to traumatizing events, such as disasters [19]. For instance, Ge and coworkers [29] found that closeness with mothers moderated the effects of both personal and family negative life events on subsequent depressive symptoms in a sample of adolescents. Based on these premises, it seems reasonable to predict that parental support could moderate the association between maternal anxiety and children’s stress to the extent that higher levels of support weaken the relationship between the variables. 

On the other hand, social (and, specifically in the present case, parental) support might not always be functional. Literature suggests that anxious and/or stressed mothers might interact less effectively with their children [30,31] and exhibit over-aroused, fearful, and vigilant behavior. Moreover, they grant less autonomy to their children and are usually less warm and more critical and catastrophizing than their healthy counterparts [32]. In this sense, anxious parents may be more likely to over-protect their children due to their own cognitive bias towards the threat, an increased perception of danger, and elevated sensitivity to their child’s distress [33]. This may convey to children the message that the world is not safe and that they will not be able to face its challenges. This form of exaggerated support (over-protection) might have been specifically relevant in the context of the pandemic, due to the objectively extraordinary (i.e., unprecedented and unknown) nature of the event, which might have exacerbated the mothers’ anxiety. Another element to be considered is the lockdown, which has caused an extensive forced closeness between parents and children. In this perspective, social support could also have had a boosting rather than a buffering effect on the relation between mothers’ anxiety and children’s stress.

Based on the two divergent perspectives on the role of social support presented above, we explored for the first time two hypotheses related to the moderation of the association between mothers’ anxiety and children’s stress in the context of the lockdown caused by the COVID-19 pandemic. Specifically, we investigated both a buffer hypothesis, according to which parental support should act as a shield and weaken the association between maternal anxiety and children’s stress, and an over-protection hypothesis, according to which “excessive” support might instead boost the aforementioned association.

### 1.3. The Present Research

As described above, previous literature showed that parents’ (and especially mothers’) mental health represents a strong predictor of their offspring’s well-being (or lack thereof). The COVID-19 pandemic has deeply impacted parents’ mental health, which, in turn, could have increased children’s stressful response to the pandemic itself. Nevertheless, parents are still regarded as one of the primary sources of social support for children. The buffering hypothesis suggests that parental support might have acted as a buffer between maternal anxiety and children’s stress. However, given the unprecedented features of the COVID-19 pandemic, we cannot exclude the opposite hypothesis (i.e., an over-protection effect that magnifies the relationship between maternal anxiety and children’s stress). Moving from the aforementioned evidence, we conducted a study to (1) test the well-acknowledged relationship between parents and children’s mental health in the novel context of the COVID-19 pandemic, and (2) answer the open question regarding the role of social support. We tested whether mothers’ anxiety is predictive of children’s stress during the COVID-19 pandemic and whether this effect is moderated by perceived parental support in a sample of dyads from regions of Italy that were highly impacted by the pandemic. It is worth noting that we collected data during the first lockdown in Italy, during which no one could exit their home (not even to exercise) except for proven needs (e.g., grocery shopping was allowed once a week by one family member). All non-essential activities were closed, and, with the suspension of in-presence teaching, education was delivered via remote learning for all grades from primary school onwards [23].

We enrolled children aged 8 to 10 years old. We focused on children from this age range because this age group has deeply suffered the effects of the lockdown [34,35]. Note that, in contrast to older children, children of this age group do not make independent use of digital technologies, therefore they are unlikely to have regular contact with peers and their social network is mainly limited to their family. To assess children’s stress, we used a reliable and widely used measure of the stressful impact of the pandemic, which can be administered from 8 years of age. 

To operationalize social support, we adopted an ecological approach, asking children how they appraised their parents’ behavior. Specifically, we asked children the extent to which they perceived support from their parents during the COVID-19 lockdown, and the relationship between mothers’ anxiety and children’s stress was examined at high and low levels of support. 

In sum, we predicted a moderation effect, such that the association between maternal anxiety and children’s stress will be either weaker (according to the buffer hypothesis) or stronger (over-protection hypothesis) at high levels of parental support.

## 2. Materials and Methods

### 2.1. Participants

The criteria for inclusion in the final sample was that both a child and the mother in the family filled out the questionnaire, answering all the constructs used in the current study. Of the 446 families that participated in the study (the call was open to both parents generally, although we expected a larger number of mothers’ responses), 95 were reached through the collaboration of primary schools in central-northern Italy, while the remaining families represented a convenience sample. The sample for final data analysis included families from which we had responses from the mothers, specifically 414 children and 395 mothers from 395 families.

While for most of the families we had the answers of one child (and the mother), for 19 families, there were two children. 229 (55.3%) of the children were female; the mean age of children was 9.44 (SD = 0.96, range 7–11 years). The mean age of the mothers was 42.84 (SD = 4.93, range 28–55 years). Of the mothers participating in the study, 12.0% were either unoccupied or housewives (*n* = 47), 8.1% were workers (*n* = 32), 2.5% worked in the healthcare system as physicians or nurses (*n* = 10), 9.9% worked in the service sector (e.g., as sales assistant) (*n* = 39), 33.8% were either administrative or commercial employees (*n* = 133), 7.1% were employed in technical professions (e.g., surveyor, hairdresser, train conductor) (*n* = 23), 23.7% worked in the educational sector (e.g., teacher) (*n* = 93), and 2.8% held manage-rial roles (*n* = 11). Two participants did not provide information about their job.

Within our sample, 6.8% had experienced at least one COVID-19 case in the family (*n* = 27) and 2.3% had one COVID-19 death in the family (*n* = 9). In terms of work, among the participants, 4.8% lost their job due to the pandemic (*n* = 19), while 38.7% shifted from face-to-face to remote working (*n* = 153).

Most of the families were living in two of the Italian regions most affected during the first wave of the COVID-19 pandemic, i.e., Emilia Romagna (72.5% of families) and Lombardy (13.8% of families) [36].

### 2.2. Procedure

The study was conducted in accordance with the Declaration of Helsinki and approved by the Ethics Committee of the University of Verona. Data were collected between April and May 2020 during the first lockdown in Italy. Mothers were reached by a unique link to a Google Form aimed at (a) presenting the research, (b) collecting informed consent for their participation and that of their child or children, (c) (for mothers) filling out their questionnaire, and (d) collecting an e-mail address to be contacted for the interview with the child/children. The interview was scheduled on an online platform (e.g., Google Meet, Skype, or Zoom, thus ensuring compliance with anti-Covid regulations), and a parent, if desired, could attend it, sitting behind the child not to condition him/her, even involuntarily. The interview was carried out by a trained interviewer who, in screen-sharing mode, read the items of the questionnaire to the child, recording his/her responses. This modality was chosen to (a) reduce error variance derived from an independent completion of the questionnaire using a technological device and (b) guarantee the understanding of the questionnaire by children, by having the interviewer available to explain eventual questions about the meaning of items.

### 2.3. Measures

*Mothers’ anxiety* was assessed with the State Anxiety Inventory (STAI-Y1) [37], which is composed of 20 items, with a response scale from 1 (*not at all*) to 4 (*very much*). Sample items were “I feel under pressure” and “I feel calm” (reverse coded). The Italian-validated version of the inventory was used [38], and the items were preceded by the headline “During the pandemic…”. A Principal Component Analysis (PCA) showed that all items loaded on one factor explained 52.5% of the variance (factor loadings ≥ 0.63). The measure had high internal consistency (Cronbach’s alpha = 0.95), and answers were averaged to create a composite score with higher values, indicating more anxiety.

*Children’s perceived parental support* was measured with a question asking the child whether their parents support them during the COVID-19 pandemic and the quarantine situation. Answers were provided on a scale ranging from 1 (*definitely no*) to 4 (*definitely yes*). The same measure was used to assess perceived peer support in a previous study with Italian primary school children [39]. Higher values indicate more perceived support. 

*Children’s stress* was assessed with the Italian version of the Children’s Revised Impact of Event Scale (CRIES-13) [40], which has been widely adopted by previously published research [25,41,42] to test Italian schoolchildren. As intended by the questionnaire, children were asked to think about how they felt during the preceding week (therefore, in the middle of the first full lockdown in Italy), and to answer 13 questions (e.g., “Do you try not to think about it?”, “Do you get easily irritable?”) on a response scale from 0 (*never*) to 3 (*often*). A PCA showed that all items loaded on one factor, which explained 26.3% of the variance. One factor loading was 0.32, while the other factor loadings were ≥0.43. The measure was internally consistent (Cronbach’s alpha = 0.76, which is in line with previous research [41]), and answers were averaged to create a composite score with higher values, indicating more stress.

## 3. Data Analysis and Results

### 3.1. Data Analysis

First, we checked skewness and kurtosis in our variables. The distribution was normal for mothers’ anxiety (skewness = 0.26, kurtosis = −0.55) and children’s stress (skewness = −0.12, kurtosis = −0.55) but not for children’s perceived parental support (skewness = −1.97, kurtosis = 3.75). 

Next, we calculated the Intraclass Correlation Coefficient (ICC) of children’s stress. Indeed, given that for some families two children were participating in the research, the data had a nested structure, with children nested in families. The ICC of children’s stress was 0.235, suggesting that the nested structure of the data should be taken into account. 

With the aim of controlling for the nested structure of the data and of taking into account the non-normality of data, the main data analysis was a regression analysis run in Mplus with the Complex command, which adjusts standard errors for the clustered structure of the data and which provides reliable estimates even when data are not normally distributed. 

Given that one item of the children’s stress measure had a low factor loading, and with the aim of ensuring the robustness of findings, we also re-ran data analysis with a 12-item composite score, excluding the item with low factor loading. 

Finally, we report descriptive statistics and bivariate Pearson correlations in Table 1 for transparency.

### 3.2. Results

**Figure 1:**
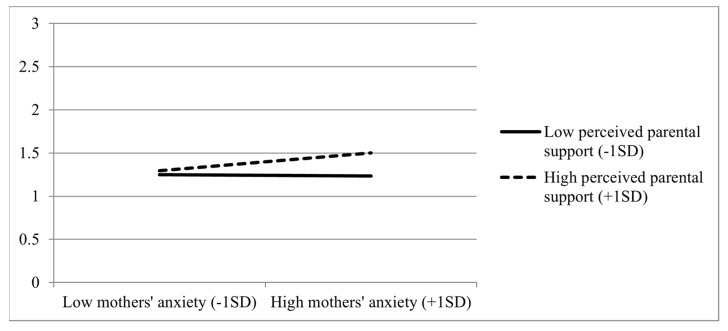
Children’s stress as a function of perceived parental support and mothers’ anxiety (*N* = 395 families, i.e., *N*_children_ = 414, *N*_mothers_ = 395).

Children’s stress regressed on (centered) mothers’ state anxiety, (centered) perceived parental support, and their interaction (Table 2), with the aim of testing whether perceived parental support moderated the associations between mothers’ anxiety and children’s stress. As hypothesized, the effect of the interaction was significant. As shown in Figure 1, mothers’ anxiety was positively associated with children’s stress only when perceived parental support was high (+1 *SD*, *b* = 0.17, *SE* = 0.06, *p* = 0.005), but not when perceived parental support was low (−1 *SD*, *b* = −0.01, *SE* = 0.05, *p* = 0.816).

The results pattern did not change when we re-run the regression analysis with the 12-item children’s stress composite score, including only items with factor loadings above 0.40. 

## 4. Discussion

We aimed to test whether mothers’ anxiety was associated with children’s stress during the COVID-19 pandemic and whether this effect was moderated by perceived parental support, by putting to test two possible and opposite moderation effects, namely a buffer hypothesis vs. an over-protection hypothesis. The COVID-19 pandemic has caused major psychological consequences for the well-being of both children and their parents [25]. Children have been particularly exposed to the negative psychological effects of COVID-19 due to home confinement, school closure, lack of in-person contact with classmates, friends, and teachers, and limitations of their personal space at home. On the one hand, children are especially sensitive to negative events and the dysfunctional feelings expressed by relevant others; on the other hand, they could have experienced more pandemic-related anxiety because of their limited understanding of the outbreak and access to coping strategies [43]. Moreover, children are less able than adults to control the negative aspects of their thoughts [44] and to communicate their feelings [45].

Despite the children in our study not showing clinical levels of stress (potentially because they were tested during the very first lockdown, which was highly stressful for adults but possibly not understood by children yet), it was still important to analyze the role of parents in determining the psychological effects of the lockdown. During the quarantine, parents have necessarily been more strictly involved in their children’s everyday activities and become their most enduring companions and supporters [46,47]. In such a situation, perceiving that the parent is distressed and unable to cope with events can be frightening for the child. In contrast with the buffering hypothesis, our results showed that mothers’ anxiety was positively associated with children’s stress only when perceived parental support was high. This counter-intuitive result might be explained via the so-called helicopter parenting effect. “Helicopter parenting” is a term used to describe a parent’s tendency to hover over their offspring [48,49]. Helicopter parents perceive (at least temporarily) the world as extremely dangerous; therefore, they can get extremely anxious and exhibit behaviors such as over-protection or over-control, which model anxious behavior in children [50]. In this sense, children are over-exposed to their parents’ anxiety, which may lead them to feel similarly threatened and to cope in an avoidant manner. 

Over-protective and anxious mothers interact less sensitively with their children [30] and tend to exhibit over-aroused, fearful, or withdrawn behavior. They can limit the autonomy of their children and be less warm as well as more critical and catastrophizing [32]. Moreover, they are more likely to model a fearful cognitive style for children, putting them at risk for anxiety-related issues as well [51,52]. A possible interpretation of our results is that during the COVID-19 pandemic, some mothers developed over-protective attitudes to secure their children. Such an over-protective attitude, instead of shielding children against mothers’ anxiety, may have made such anxiety especially evident, resulting in a highlighted positive association between mothers’ anxiety and children’s stress. In other words, children might have been overwhelmed by their mother’s anxiety due to disproportionate supporting behavior which drove attention to anxiety rather than away from it. If this interpretation of data is correct, our study might represent the first evidence of the helicopter parenting effect in the context of COVID-19. Indeed, this result is in line with research identifying mothers’ anxiety as the most important predictor of children’s mental health [6] and highlights the critical role of the parent in children’s well-being [53,54]. 

The pandemic has caused severe psychological difficulties for adults. Many reported distress, irritability, restlessness, anger, sadness, worry, and mental health issues, such as depression and anxiety. Possibly, stress experienced during the pandemic interfered with parents’ ability to take care of their children. Stressed parents can manifest difficulties in understanding children’s needs and responding sensitively [55]; they may be too overwhelmed to find appropriate ways to support their children and properly address their questions and fears [56]. When children do not find responsive answers to their preoccupations, they may show increased distress, often expressed as emotional and behavioral problems [23]. Importantly, mothers may be unable to hide their anxiety. When they over-protect while trying to support, they may evidence their anxiety, thus increasing the chance of it spilling over.

We believe this study has important novelties. First, it extends literature showing an association between mothers’ anxiety and children’s stress in the context of the COVID-19 pandemic. In contrast with preliminary evidence [23,24,25], it employs children’s self-reported measures via face-to-face interviews, rather than relying on parents’ perceptions. Second, it suggests that the buffer effect might not apply to parental support in the specific context of the COVID-19 pandemic, during which mothers’ anxiety might have determined a helicopter parenting style that further increased children’s stress. This evidence shows the importance of mothers’ responses but also their potential dangerousness when they imply over-protection rather than secure scaffolding.

We acknowledge some limitations. First, the data are correlational and, therefore, no certain causal conclusion can be drawn from them. It cannot be excluded that the variables we tested could be associated in different ways (e.g., children’s stress could be the precursor of high parental support), or even in a circular way. However, considering previous literature, we believe it unlikely that the direction of causality is reversed, even if longitudinal assessments would provide greater confidence. Second, children’s responses may have been biased by the fact that many mothers attended their interviews, with children that might have strategically changed their responses to meet unexpressed mothers’ expectancies. Given these motivations, we can only interpret from our moderation analyses that the children may have perceived an excessive and intrusive presence, leaving open the possibility of alternative interpretations or that some children were offered secure support. Note that, in the latter case, our results emerged even if only a part of the children in the sample experienced mothers’ over-protection, which makes them more noteworthy. Future studies might find a way to address the different content of parental support, using it as a moderator of the effect we found. Another limitation pertains to the use of a single item to measure parental support, which was also meant to keep the children’s (who already spent many hours on their computers for remote learning) screen time to the minimum. Even if this measure was successfully used with a similar sample [39], future studies might employ validated measures of social support. A related issue about measures refers to the mismatch between the target of the measure of anxiety (mother) and perceived support (parents). We reasoned that in such a problematic situation, asking children about support from the two parents separately may have implicitly suggested differences in the parental approach, raising ethical problems. At the methodological level, responses may have been biased by the fact that, in most of the interviews, the mother was present, causing social desirability issues in children’s responses. Possibly, results would have been stronger with matched measures. In any case, we caution on the interpretation of our findings in light of these limitations.

## 5. Conclusions

On a theoretical level, our study further investigates the moderating role of social support in the relationship between an environmental stressor (in this case, maternal anxiety) and the child’s mental health (here operationalized as COVID-19-related stress), arriving at unexpected but interesting results. Specifically, in emergency contexts, emotional support expressed by mothers might have paradoxical and unwanted negative consequences for children’s stress when maternal anxiety is pervasive. These results could be explained in light of the specific context of the COVID-19 pandemic and the consequent lockdown, which could have altered and magnified the offer of social support by parents. However, this interpretation is based on correlational data, and will therefore need to be validated by future studies. At the practical level, our findings stimulate reflection on the impact of the pandemic on the mental health of children, and on the most appropriate ways of supporting it. Indeed, public institutions primarily focused on physical health, recommending steps for the prevention and containment of the disease, while the impact on mental health remained almost undiscussed. However, as we derive from this and many other studies, the role of the parents in determining children’s well-being is of the highest relevance [53,54]. To prevent children’s socio-emotional problems and promote their well-being in the face of adversities, public institutions should therefore recognize the importance of supporting parents first. Based on our findings’ interpretation, an example of good practice could be providing parents with knowledge about how to contain their own and their children’s anxiety by discussing fears and negative emotions [46]. This would help to empower parents, safeguard children, and potentially reduce the social costs of the pandemic at many levels.

## Figures and Tables

**Table 1:** Means, standard deviations, and Pearson correlations between variables.

	Mean (*SD*)	1	2
1. Mother’s anxiety	2.16 (0.61)	-	
2. Children’s perceived parental support	3.60 (0.70)	0.00	-
3. Children’s stress	1.32 (0.56)	0.09 °	0.14 **

Notes. ° *p* = 0.062. ** *p* < 0.01.

**Table 2:** Regression analysis predicting children’s stress.

	Children’s Stress
Intercept	1.32 (0.02) ***
Mother’s anxiety	0.08 (0.04) °
Children’s perceived parental support	0.11 (0.04) ***
Mothers’ anxiety × children’s perceived parental support	0.13 (0.05) **
***R* ^2^**	0.04 *

Notes. Unstandardized coefficients (and standard errors) are reported. ° *p* = 0.067. * *p* < 05. ** *p* < 0.01. *** *p* ≤ 0.001. In bold is the coefficient of determination (i.e., the proportion of variation in the dependent variable predicted by the independent variables).

## Data Availability

The data that support the findings of this study are available from the corresponding author, C.A., upon reasonable request.
